# Association between air pollution and COVID-19 mortality and morbidity

**DOI:** 10.1007/s11739-021-02834-5

**Published:** 2021-10-12

**Authors:** Karolina Semczuk-Kaczmarek, Anna Rys-Czaporowska, Janusz Sierdzinski, Lukasz Dominik Kaczmarek, Filip Marcin Szymanski, Anna Edyta Platek

**Affiliations:** 1 1st Department of Cardiology, Medical University of Warsaw, Banacha 1A St., 02-097 Warsaw, Poland; 2 Department of Medical Informatics and Telemedicine, Medical University of Warsaw, Warsaw, Poland; 3 Faculty of Building Services, Hydro and Environmental Engineering, Warsaw University of Technology, Warsaw, Poland; 4 Departament of Civilization Diseases, Faculty of Medicine, Collegium Medicum, Cardinal Stefan Wyszyński University in Warsaw, Warsaw, Poland; 5 Department of General and Experimental Pathology, Medical University of Warsaw, Warsaw, Poland

**Keywords:** Air pollution, SARS-CoV-2, COVID-19, Cardiovascular disease

## Abstract

Coronavirus disease (COVID-19) pandemic is affecting the world unevenly. One of the highest numbers of cases were recorded in the most polluted regions worldwide. The risk factors for severe COVID-19 include diabetes, cardiovascular, and respiratory diseases. It has been known that the same disease might be worsened by chronic exposure to air pollution**.** The study aimed to determine whether long-term average exposure to air pollution is associated with an increased risk of COVID-19 cases and deaths in Poland. The cumulative number of COVID-19 cases and deaths for each voivodeship (the main administrative level of jurisdictions) in Poland were collected from March 4, 2020, to May 15, 2020. Based on the official data published by Chief Inspectorate of Environmental Protection voivodeship-level long-term exposure to main air pollution: PM_2.5_, PM_10_, NO_2_, SO_2_, O_3_ (averaged from 2013 to 2018) was established. There were statistically significant correlation between COVID-19 cases (per 100,000 population) and annual average concentration of PM_2.5_ (*R*^2^ = 0.367, *p *= 0.016), PM_10_ (*R*^2^ = 0.415, *p *= 0.009), SO_2_ (*R*^2^ = 0.489, *p *= 0.003), and O_3_ (*R*^2^ = 0.537, *p *= 0.0018). Moreover, COVID-19 deaths (per 100,000 population) were associated with annual average concentration of PM_2.5_ (*R*^2^ = 0.290, *p *= 0.038), NO_2_ (*R*^2^ = 0.319, *p *= 0.028), O_3_ (*R*^2^ = 0.452, *p *= 0.006). The long-term exposure to air pollution, especially PM_2.5_, PM_10_, SO_2_, NO_2_, O_3_ seems to play an essential role in COVID-19 prevalence and mortality. Long-term exposure to air pollution might increase the susceptibility to the infection, exacerbates the severity of SARS-CoV-2 infections, and worsens the patients’ prognosis. The study provides generalized and possible universal trends. Detailed analyzes of the phenomenon dedicated to a given region require taking into account data on comorbidities and socioeconomic variables as well as information about the long-term exposure to air pollution and COVID-19 cases and deaths at smaller administrative level of jurisdictions (community or at least district level).

## Introduction

Coronavirus disease (COVID-19) has appeared in Wuhan (China) in December 2019 [1]. Owing to the rapid geographic transmission of severe acute respiratory syndrome coronavirus 2 (SARS-CoV-2), the World Health Organization declared the outbreak of a Public Health Emergency of International Concern on January 30, 2020. Up to May 21, 2020, more than 4.89 million cases of COVID-19 were reported, resulting in 323 256 deaths in 216 countries [2]. The symptoms caused by the SARS-CoV-2 infections can range from mild (even asymptomatic) through fever, dry cough, fatigue, shortness of breath, up to pneumonia, and acute respiratory distress syndrome (ARDS). The clinical manifestation depends on the patient’s age, physical condition, and comorbidities. The Centers for Disease Control and Prevention ‘s list of risk factors for severe COVID-19 include diabetes, heart diseases, and chronic airway diseases (i.e., asthma, lung cancer, and chronic obstructive pulmonary disease) [3]. Most of those risk factors may be triggered by chronic exposure to air pollution.

Air pollution consists of volatile organic compounds, small particulate matter (PM_2.5_, PM_10_), and gases [nitric dioxide (NO_2_); carbon monoxide (CO); ground-level ozone (O_3_), sulfur dioxide (SO_2_)] derived from industrial emissions, vehicular traffic, or indoor pollutants. The larger mass of chemical compounds in air pollution is made of semi-volatile or nonvolatile compounds, in particular, the group of polycyclic aromatic hydrocarbons (PAHs). It is worthy to underline that exposure to PAHs (emissions from motor vehicles, industrial plants, power generation plants, waste incinerators, and open burning) has been linked with lung cancer, cardiovascular disease and poor fetal development. Thus, if such data are recorded commonly, it is recommended to use it. In the described case, the most complete and available data concerned the parameters listed at the beginning of the paragraph. It has been estimated that every year ≈ 7 million people worldwide die from exposure to polluted air [4]. Almost half of them will die of ischemic heart disease or stroke attributed to exposure to air pollution. Making air pollution more significant than any other major modifiable cardiovascular risk factors (smoking, hypertension, hyperlipidemia, and diabetes mellitus) [4]. For these reasons, we hypothesize that air pollution may worsen the prognosis of COVID-19 patients by exacerbating underlying cardiovascular or respiratory diseases and suppressing immune responses [5].

Poland is one of the most polluted countries in the European Union (EU). Out of the 50 most polluted cities in the EU, 33 are in Poland. Poland's main problem is excessive concentration of tropospheric ozone in summer and PM_10_ and benzo(a)pyrene in winter [6]. It has been estimated that due to exposure to air pollution, the life expectancy of an average Polish citizen is shortened by around 9 months, and 48 thousand people die prematurely every year due to air pollution [7, 8]. The current study aimed to assess if long-term average exposure to air pollution is associated with an increased risk of COVID-19 cases and deaths in Poland.

In the study, we take into account, the administrative division of Poland for sixteen voivodships. Voivodships is the highest level administrative division of Poland, corresponding to a province or state in many other countries.

## Methods

### COVID-19 cases and deaths in Poland

The official data of SARS-CoV-2 infections and deaths by voivodeships in Poland are published daily by the Polish Ministry of Health [9]. All cases are diagnosed as positive based on the polymerase chain reaction tests for SARS-CoV-2. We collected the cumulative number of cases and deaths for each voivodeship in Poland from March 4, 2020 (first confirmed COVID-19 case in Poland) up to May 15, 2020. Individual-level data of COVID-19 deaths and cases in Poland are currently not available.

### Exposure to air pollution

The effect of chronic exposure tends to be stronger than relationships with short-term exposure [10, 11]. Therefore, we calculated voivodeship-level long-term exposure to main air pollution: PM_2.5_, PM_10_, NO_2_, SO_2_, O_3_ (averaged from 2013 to 2018). Chief Inspectorate of Environmental Protection publishes the official data about exposure, and they are the results of measurements by air pollution monitoring stations throughout the 16 voivodeships in Poland. These stations belong mainly to the Chief Inspectorate of Environmental Protection, as well as to research institutes and other organizations cooperating within the Chief Inspectorate of Environmental Protection. The currently available official data include the annual average level of PM_2.5_, PM_10_, NO_2_, SO_2_, O_3_, updated yearly from 2013 to 2018 [12].

According to the Reports of the State of Environment in Poland published by Chief Inspectorate of Environmental Protection upper limit of the annual average concentration of air pollution in Poland are dependent on the kind of pollution. Thus, upper limit for annual average concentration of PM_2.5_, PM_10_, NO_2_, SO_2_, O_3_ are 25 µg/m^3^, 40 µg/m^3^, 40 µg/m^3^, 125 µg/m^3^, 120 µg/m^3^, respectively (based on the European Parliament and Council Directive 2008/50/EC) [12]. Reports of the State of Environment in Poland provide the number of days with O_3_ concentration exceeding the upper limit (120 µg/m^3^).

### Statistical methods

Voivodeship-level COVID-19 prevalence rates were defined as the ratio of COVID-19 incidence to voivodeship level population size. For further detailed analyses, COVID-19 cases (per 100,000 population) and deaths (per 100,000 population) in each voivodeship were calculated. The association between all assessed parameters and COVID-19 prevalences and mortality was assessed using Spearman’s correlation. Although individual-level data would have allowed a more rigorous statistical analysis, individual-level data on COVID-19 death is currently unavailable. Forward stepwise multivariate logistic regression models were created to identify the independent predictors of COVID-19 cases and deaths. A *p* value < 0.05 was considered statistically significant. Statistical processing of data was made using SPSS v. 21 software (SPSS Inc., Chicago, USA).

## Results

As of May 15, 2020, there were 18,016 laboratory-confirmed COVID-19 cases and 907 deaths. The highest number of COVID-19 cases was in Silesian (*n *= 4994 cases), and Mazovian Voivodeship (*n *= 2930); the lowest was in Lubusz (*n *= 92 cases) and Warmian–Masurian Voivodeship (*n *= 170 cases). Similarly, the highest number of COVID-19 death was in Mazovian (*n *= 231 deaths) and Silesian Voivodeship (*n *= 172); the lowest was in Lubusz (*n *= 0) and Warmian–Masurian Voivodeship (*n *= 1 case).

From 2013 to 2018, the highest average concentrations of PM_2.5_ and PM_10_ were in Silesian (30.27 µg/m^3^ and 42.35 µg/m^3^, respectively), Lesser Poland (30.87 µg/m^3^ and 40.18 µg/m^3^, respectively) and Lodz Voivodeship (26.71 µg/m^3^ and 37.99 µg/m^3^, respectively). In addition, these values exceed the upper limit for annual average concentration on PM_2.5_ and PM_10_. The lowest average concentration of PM_2.5_ and PM_10_ was in Warmian–Masurian (15.78 µg/m^3^ and 25.45 µg/m^3^, respectively), Pomeranian (16.88 µg/m^3^ and 23.66 µg/m^3^, respectively), West Pomeranian Voivodeship (17.22 µg/m^3^ and 24.61 µg/m^3^, respectively).

The highest concentration of NO_2_ was noted in Lesser Poland (40.25 µg/m^3^), SO_2_ concentration in Silesian (55.53 µg/m^3^), and the highest number of days with O_3_ concentration exceeding the upper limit was in Lower Silesia Voivodeship (8 days). Podlaskie Voivodeship had the lowest concentration of NO_2_ (11.73 µg/m^3^), SO_2_ (10.21 µg/m^3^), and the lowest number of days with O_3_ concentration exceeding the upper limit 30 days).

**Fig. 1:**
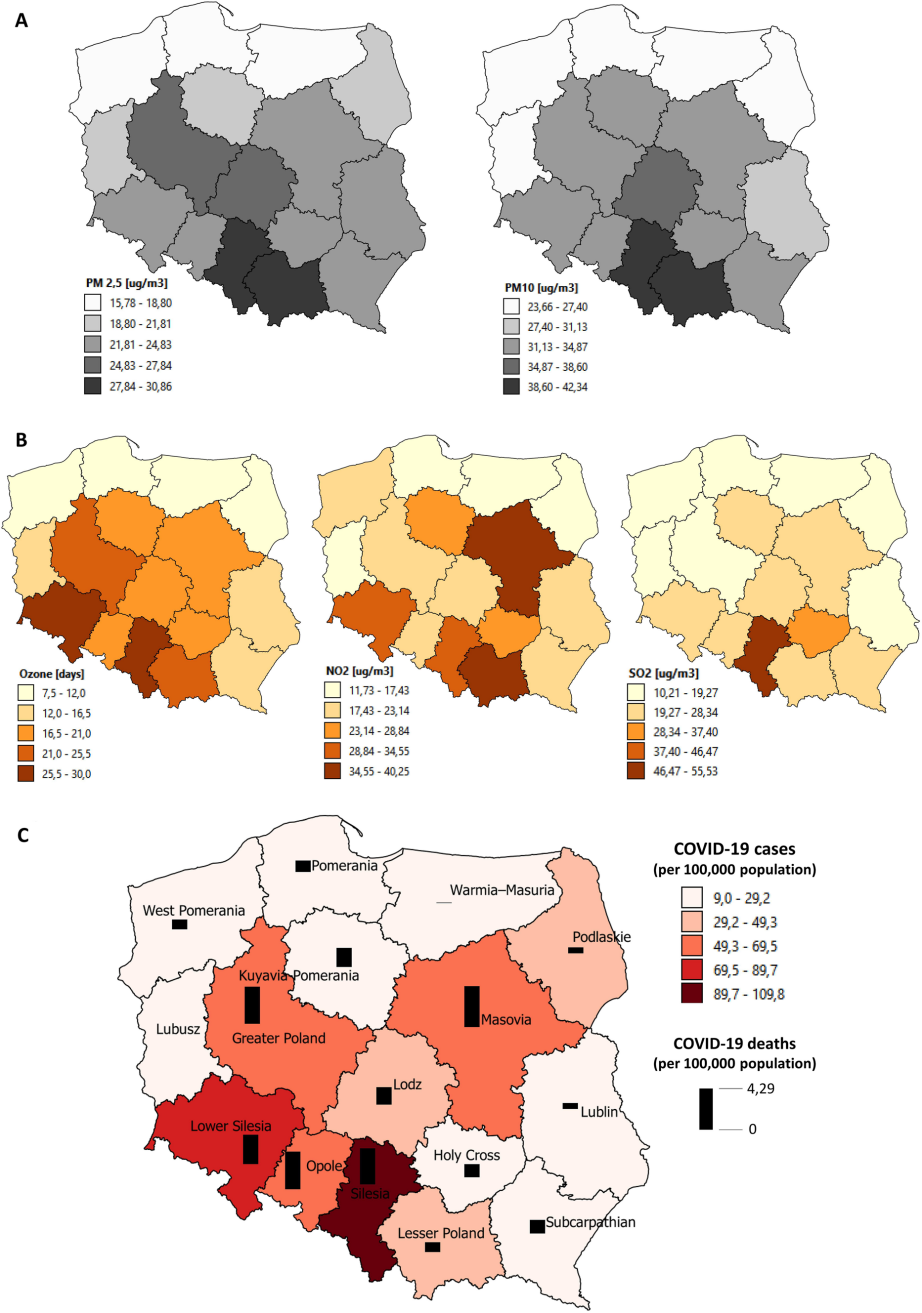
Summary of the average concentration of air pollution, COVID-19 cases, and death in voivodeships in Poland (**A** concentration of PM_2.5_; PM_10_; **B** concentrations of O_3_, NO_2_, SO_2_; C, COVID-19 cases and death per 100,000 population)

We have observed statistically significant correlation between COVID-19 cases (per 100,000 population) and annual average concentration of PM_2.5_ (coefficient of determination, *R*^2^ = 0.367, *p *= 0.016), PM_10_ (*R*^2^ = 0.415, *p *= 0.009), SO_2_ (*R*^2^ = 0.489, *p *= 0.003), and O_3_ (*R*^2^ = 0.537, *p *= 0.0018). Moreover, there was a significant correlation between COVID-19 deaths (per 100,000 population) and PM_2.5_ (*R*^2^ = 0.290, *p *= 0.038), NO_2_ (*R*^2^ = 0.319, *p *= 0.028), O_3_ (*R*^2^ = 0.452, *p *= 0.006) concentrations (Table 1). Summary of average concentration of air pollution, COVID-19 cases, and death (per 100,000 population) in voivodeships in Poland is presented in Fig. 1.

**Table 1:** Correlation between COVID-19 cases and deaths (per 100,000 population) and annual average concentration of air pollution

Parameter	Value (mean ± SD)	COVID-19 cases (per 100,000 population)		COVID-19 deaths (per 100,000 population)	
		*R*^2^	*p*	*R*^2^	*p*
PM_2.5_ (µg/m^3^)	22.90 ± 4.49	0.367	0.016	0.290	0.038
PM_10_ (µg/m^3^)	31.40 ± 5.48	0.415	0.009	0.252	0.056
SO_2_ (µg/m^3^)	21.70 ± 10.87	0.489	0.003	0.169	0.127
NO_2_ (µg/m^3^)	23.58 ± 7.82	0.262	0.051	0.319	0.028
O_3_ (the number of days with O_3_ concentration exceeding the upper limit (120 µg/m^3^)	17.16 ± 6.62	0.537	0.0018	0.452	0.006

*R*^2^ coefficient of determination, *SD* standard deviation

## Discussion

The observed correlation between exposure to air pollution and COVID-19 cases and deaths in Poland are consistent with previously published studies describing these associations in other countries [13]. The first nationwide study of the relationship between historical exposure to air pollution exposure and the COVID-19 death rate was conducted in the United States by Wu et al. [13]. They analyzed data for county-level long-term exposure to PM_2.5_ (averaged from 2000 to 2016) on county-level COVID-19 deaths. They found that an increase of at least 1 µg/m^3^ in long-term average PM_2.5_ was associated with an 8% increase in the COVID-19 death rate—(RR 1.08; 95% CI 1.02–1.15). They also observed that other factors might be predictors of higher risk of COVID-19 mortality rate—population density, rate of hospital beds, days since first COVID-19 case reported and less obvious—median household income, less than high school education, and Black Americans. The mechanism that may explain the relationship between air pollution and viral outcomes is that PM_2.5_ exposure is associated with many of the cardiovascular and respiratory comorbidities that increase the severity and risk of death in COVID-19 patients [14]. The immune responses might be suppressed by exposure to pollution, which leads to worse prognosis [15].

At the early stage of the pandemic, Italy was the country with the highest number of COVID-19 infections in Europe [16]. The outbreaks focused in North Italy—Po Valley, and the cities of Lodi, Cremona, and Bergamo. Conticini et al. noted that these cities are in the five Italian cities with the highest pollution emissions due to a high density of factories, traffic, and intensive agriculture [16]. Specific topography (plain surrounded by the Alps), climatic features (frequent episodes of climatic inversion) cause the stagnation of pollutants. The concentrations of PM_2.5_ over this region reached very high values, which are similar to those characterizing the Hubei province in China, where the first COVID-19 cases were recognized.

In addition, the case fatality for COVID-19 in North Italy was 12%, and was significantly higher as compared to 4.5% in the rest of Italy [16]. The authors suggest that observed high prevalence and COVID-19 mortality might be secondary to long-term exposure to air pollution. Previous studies report that long-term exposure to air pollution impairs cilia and upper airways defenses, promotes a chronic inflammatory state, increases the risk of chronic respiratory disease [11]. Another analysis also confirmed that long-term air-quality data (NO_2_, O_3_, PM_2.5,_ and PM_10_) significantly correlated with cases of COVID-19 in up to 71 Italian provinces [17].

Another study found that COVID-19 mortality was associated with NO_2_ concentration [18]. It has been observed that 78% of fatality cases (*n *= 3487) occurred in North Italy and central Spain regions with the highest NO2 concentration. In the previous studies, elevated exposure to NO_2_ was connected with hypertension, cardiovascular, chronic obstructive pulmonary disease (COPD), and lung injury [18]. It was also described the destroying impact of long-term NO_2_ exposure on epithelial cells in the lung and promoting the synthesis of proinflammatory cytokines from airway epithelial cells.

Another research group using annual indices of air quality analyzed the correlation between air pollution and SARS-CoV-2 morbidity and mortality [19]. It found significant positive correlations between air quality variables and COVID-19 cases, with higher rates of COVID-19 infection in areas with high NO_2_ and CO concentration. Higher mortality was also correlated with high PM_2.5_, CO, and NO_2_ concentration [19]. Similarly, a cross-sectional analysis conducted by Yao et al. showed positive correlations between NO_2_ pollution levels and COVID-19 transmission rates in the cities in Hubei province, China [20]. In addition, they observed that increased concentrations of PM_2.5_ and PM_10_ were linked to higher death rates from COVID-19 (*p *= 0.011 and *p *= 0.015, respectively) [21]. Nevertheless, some researchers supposed that the atmosphere rich in air pollutants might promote a longer permanence of the viral particles in the air and facilitate the spreading of SARS-CoV-2 [22].

The observed association might be linked to low-grade pulmonary damage and inflammation caused by air pollution [23]. There is a hypothesis that long-term exposure to air pollution might increase the susceptibility to the infection and impair pulmonary defense mechanisms [23, 24]. Some hypothesize that exposure to air pollution and SARS-CoV-2 is a “double-hit” to the lungs and lead to acute lung injury [8]. PM_2.5_ penetrates the peripheral air spaces and, through the interaction with the lung renin–angiotensin system (RAS) may facilitate viral infection [8]. It is described that angiotensin-converting enzyme 2 (ACE-2) expressed at the alveolar level is a co-receptor for the viral entry of SARS-CoV-2 through interaction with viral spike proteins (VPS) [25]. Patients chronically exposed to high levels of PM_2.5_ overexpress ACE-2. High pulmonary expression of ACE-2 correlates with susceptibility to SARS-CoV-2 infection. SARS-CoV-2 binding to ACE-2 might induce deficient anti-inflammatory action leading to acute lung injury [25].

### Limitations

No studies were published concerning the impact of air quality in Central Europe on COVID-19 cases. Nevertheless, our study has some limitations. First, similarly to other studies nationwide, individual-level COVID-19 outcome data are unavailable at this time. At the time of preparing this study commonly available was the data on the number of COVID-19 cases and deaths at voivodships level (the main administrative level of jurisdictions in Poland). Such the data enable the derivation of important generalized and possible universal trends. We suggest that the data from smaller administrative level of jurisdictions (community or at least district level) will deliver more accurate information about the impact of local long-term exposure to air pollution and COVID-19 cases and deaths. This type of study should be designed in the future when more detailed information will be available. Detailed analyzes of the COVID-19 pandemic phenomenon dedicated to a given region requires taking into account data on comorbidities and socioeconomic variables as well as information about the long-term exposure to air pollution and COVID-19 cases and deaths at smaller administrative level of jurisdictions (community or at least district level). Secondly, confirmed COVID-19 cases probably are a supposed percent of the exact SARS-CoV-2 infection incidence rate because, in most cases, asymptomatic patients or patients with mild symptoms were not tested and remain unidentified [26]. Thirdly, highly polluted areas are characterized by higher rates of human interaction and international travelers, which facilitates the spreading of SARS-CoV-2 infection. An increase in COVID cases for 100,000 population might lead to a proportional increase in COVID mortality for 100,000 population. In our opinion, it is crucial to underline that our study and mentioned above articles describe the association between COVID-19 prevalence, mortality, and air pollution. We proposed that other factors widespread in these densely populated and highly industrialized areas like long-term exposure do air pollution, especially PM_2.5_, NO_2_, and O_3_ play an important role in COVID-19 mortality**.** We observed, that the highest number of COVID-19 cases and deaths per 100,000 populations were recorded in the most polluted voivodeships in Poland: Silesian, Lower Silesian, Masovian, Opole, Lodz, Greater Poland Voivodeships, whereas the lowest number was noted in voivodeships with better air quality: Lubusz, Warmian–Masurian, Podlaskie Voivodeships. We concluded that long-term exposure to air pollution, especially PM2.5, PM10, SO2, NO2, O_3_ seems to play an essential role in COVID-19 prevalences and mortality. The obtained results indicate the importance of further research using interdisciplinary data recorded at the lowest levels of regions division.

Last but not least, regression models (as used in this paper) in epidemiological research that involves air pollution are not perfect tools for assessing whether reducing exposure to air pollution will reduce the risk of harm to human [27]. This role is assigned for the traditional scientific method based on the testing predictive generalizations against data. It is worthy to underline that science should be completed (not substituted) by regression models [27].
